# Adoption and scale-up of the cardiovascular Polypill: a realist institutional analysis

**DOI:** 10.1093/heapol/czac088

**Published:** 2022-10-22

**Authors:** Seye Abimbola, Hueiming Liu

**Affiliations:** School of Public Health, Faculty of Medicine and Health, The University of Sydney, Camperdown, NSW, Australia; The George Institute for Global Health, University of New South Wales, Sydney, NSW, Australia

**Keywords:** Innovation, governance, institutions, realist, scale-up, cardiovascular disease, fixed-dose combinations, single-pill combinations, Polypill

## Abstract

Efforts to promote the adoption and scale-up of health system innovations must contend with the existing institutional context. But there are no commonly used frameworks to ensure that the insights of actors involved in such institutional efforts connect to one another. To test and modify a potential framework—the ‘four-by-four’ framework—we interviewed researcher-entrepreneurs involved in the unfolding story of the cardiovascular Polypill. The framework has four types/levels of institutions that affect adoption and scale-up: (1) informal institutions (L1, e.g. social norms), (2) formal institutions (L2, e.g. government policies and regulation), (3) organizational structures (L3, e.g. organizational boards and mission) and (4) everyday exchange (L4, e.g. service delivery), vis-à-vis four potential entrepreneurial strategies in response: (1) abide by existing institutions, (2) evade them, (3) alter them and/or (4) exit entrepreneurial action. Using this framework, we conducted a realist-informed analysis to understand how context (i.e. institutions) and mechanism (i.e. entrepreneurial strategies) influence each other to shape outcomes (i.e. adoption and scale-up). We found that researcher-entrepreneurs began with efforts to abide with existing institutions but encountered institutional obstacles at each level. Efforts to abide were followed by seeking to evade and/or alter unfavourable institutions, with greater success evading and/or altering lower (L3 and L4) than upper (L1 and L2) institutions. Exit considerations followed the failure of the evade or alter strategy. Shifts between strategies were propelled by ‘learning’. The ‘four-by-four’ framework can be used as a scaffold to generate narratives of adoption or scale-up efforts, a sensitizing tool to prospectively map out contingencies and a matrix to synthesize narratives and experiences across multiple innovations or settings. Used in these ways, the ‘four-by-four’ framework can help to optimize the transferability and cumulation of insights on how to promote the adoption and scale-up of health system innovations.

**Key messages**

- Adoption and scale-up of health system innovations is a goal that poses a perennial challenge for researchers, policymakers and programme implementers. While frameworks exist to inform and analyse the adoption and scale-up of (especially technological) innovations, those frameworks neither place governance (and, by extension, institutional uncertainty) at the centre of consideration nor do they focus on the crucial role of the policy and practice entrepreneurs seeking to institutionalize an innovation.
- Using the example of an innovation (the cardiovascular Polypill) we proposed a ‘four-by-four’ framework to fulfil these needs. That is, four types/levels of institutions that may affect adoption and scale-up: (1) informal institutions (L1, e.g. social norms), (2) formal institutions (L2, e.g. government policies and regulation), (3) organizational structures (L3, e.g. organizational boards and mission) and (4) everyday exchange (L4, e.g. service delivery), vis-à-vis four potential entrepreneurial strategies in response: (1) abide by existing institutions, (2) evade them, (3) alter them and/or (4) exit entrepreneurial action.
- This ‘four-by-four’ framework can be used as a scaffold to generate narratives of adoption or scale-up efforts, as a sensitizing tool to prospectively map out contingencies and as a matrix to synthesize narratives and experiences across multiple innovations and different settings. Used in these various ways, the ‘four-by-four’ framework can help to optimize the transferability and cumulation of insights on how to promote the adoption and scale-up of health system innovations—something that is currently lacking.

## Introduction

How to optimize the adoption and scale-up of health system innovations is a recurrent concern in local and global public health discussions (Yamey, 2012; Greenhalgh *et al*., 2017b; Petersen *et al.*, 2019; Calibo *et al.*, 2021; Chamberlain *et al.*, 2021; Osei Afriyie *et al.*, 2021). However, such (policy, practice and research) discussions are often conducted for different innovations independent of each other, therefore limiting the potential for cross-learning. The discussions have also been conducted using different frameworks and terminologies. Insights that, on closer examination, may speak to one another may not immediately appear related or seem relevant more broadly. Unfortunately, these insights are not used optimally by policy or practice entrepreneurs (including researchers) who seek to institutionalize innovations in various settings. This fragmentation of discourse is evident in recent studies documenting and theorizing (interactions among) factors that enable or constrain the adoption and scale-up of different health system innovations (Yamey, 2012; Greenhalgh *et al.*, 2017a,b; Abimbola and Keelan *et al.*, 2019; Nanyonjo *et al.*, 2019; Petersen *et al.*, 2019; Bulthuis *et al.*, 2020; Calibo *et al.*, 2021; Chamberlain *et al.*, 2021; Osei Afriyie *et al.*, 2021).

Another challenge to optimizing the adoption and scale-up of health system innovations relates to what is deemed an ‘innovation’—often tangible, technological or even electronic. There is a much greater intellectual investment in consolidating insights on the adoption and scale-up of such innovations, e.g. through the non-adoption, abandonment, scale-up, spread and sustainability (NASSS) framework, which was developed in 2017 based on in-depth experience of implementing technological innovations in high-income countries (HICs) (Greenhalgh *et al.*, 2017a). It has been cited >1000 times. The NASSS framework highlights seven domains that influence the success of such an innovation: the illness, the technology, the value proposition, the adopter system (e.g. service providers and users), the organizations, the wider institutional and societal context and the interaction between the domains over time. Earlier, the development of the Normalization Process Theory (NPT) in 2009 was also informed by the implementation of technological innovations in HICs (May and Finch, 2009). It has been cited >1500 times. NPT proposes four constructs of what people do to ‘normalize’ a new practice: coherence, cognitive participation, collective action and reflexive monitoring.

In comparison, non-technological innovations receive less conceptual attention, therefore limiting the extent to which insights on their adoption and scale-up can cumulate. Especially innovations—both technological and non-technological—in settings where health systems are under-governed; settings in which optimal health system functioning is not a given (Abimbola *et al.*, 2017; Bylund and McCaffrey, 2017; Greenhalgh and Abimbola, 2019; Abimbola, 2020), whether in HICs or in low- and middle-income countries (LMICs). In such settings, the influence of governance (i.e. the making, changing, monitoring and enforcing the formal and informal rules that shape interactions among societal actors; Abimbola *et al.*, 2017) and institutional uncertainty (i.e. uncertainties in the existing arrangement of rules; Bylund and McCaffrey, 2017) on the adoption or scale-up of health system innovations inevitably looms large (Bylund and McCaffrey, 2017). Unlike widely used and important frameworks such as NPT and NASSS, any framework that will allow insights across innovations (of all kinds) and across settings (well-governed or under-governed) to cumulate will need to take governance (alongside institutional uncertainty) as an important starting point. The links among governance, institutional uncertainty and the adoption and scale-up of innovations have so far been under-theorized (Bylund and McCaffrey, 2017).

Consider, e.g. the following literature reviews analysing the adoption and scale-up of various health system innovations—first, an analysis of efforts to institutionalize integrated community case management into the health systems of LMICs (Nanyonjo *et al.*, 2019); second, of factors broadly influencing the scale-up of public health interventions in LMICs (Bulthuis *et al.*, 2020); third, of domains to predict and evaluate the success or failure of technology-supported health programmes across HICs (Greenhalgh *et al.*, 2017a) and fourth, of patient-facing telehealth in HICs (Abimbola and Keelan *et al.*, 2019). Each analysis highlighted the dynamic interactions among a range of factors that influence the adoption and scale-up of innovations. Using different frameworks, each categorized the interactions among the factors differently. However, there was one domain—governance (alongside institutional environment)—around which much of the others revolved. But it was framed variously as ‘wider institutional and societal context’, ‘sociocultural environment’, ‘alignment between supply and demand’, ‘leadership’, ‘politics’, ‘political will’, ‘regulation’, ‘strategy’ and ‘advocacy’ (Greenhalgh *et al.*, 2017a; Abimbola and Keelan *et al.*, 2019; Nanyonjo *et al.*, 2019; Bulthuis *et al.*, 2020). In those analyses, what may be defined as governance (and institutional environment) was also centred and subcategorized differently.

In this article, we analyse the adoption and scale-up of the cardiovascular Polypill (Wald and Law, 2003). As an innovation, the Polypill is not as tangible as a technological or electronic innovation, but also not as intangible as a new process or way of doing things like integrated community case management. By being such a liminal innovation—with ongoing adoption and scale-up efforts and uncertainties across LMICs and HICs—the cardiovascular Polypill offers conceptual insights that cut across a broad range of innovations and settings. While the idea of combining antihypertensives, a statin and aspirin to prevent cardiovascular disease was first proposed in 2001 (World Health Organization and Wellcome Trust, 2002), the term ‘Polypill’ was coined by Wald and Law in 2003 to describe a strategy of using a single-pill combination of drugs for population-level control of cardiovascular disease risk factors (Wald and Law, 2003). Their proposed Polypill combines six drugs (three antihypertensives, a statin, aspirin and folic acid) to reduce high blood pressure, high blood cholesterol, high serum homocysteine and platelet dysfunction. Wald and Law suggested through a modelling study that if administered ‘to everyone aged 55 and over’, the Polypill ‘would prevent 88% of heart attacks and 80% of strokes’ (Wald and Law, 2003)—based on the assumption that combining the drugs will have additive effects on patient outcomes.

The proposal immediately divided opinions (Smith, 2009). It has continued to do so. The Polypill was seen as a promising solution if its effectiveness could be proven in randomized controlled trials (RCTs) and if its component drugs could be the low-cost generic versions (Rodgers, 2003). On the other hand, the Polypill was criticized as an unnecessary over-medicalization of a public health issue (Roy *et al.*, 2017). In the 20 years since the cardiovascular Polypill (excluding folic acid whose effectiveness was not confirmed in standalone RCTs) was first proposed, RCTs have repeatedly demonstrated the feasibility and the efficacy of versions of the Polypill (Elley *et al.*, 2012; Webster *et al.*, 2016; Bahiru *et al.*, 2017; Kandil *et al.*, 2022; Mohamed *et al.*, 2022)—to improve outcomes and reduce risk factors such as high blood pressure and cholesterol. Many RCTs on the cardiovascular Polypill have been conducted in single- and multi-country studies—in Africa (Tunisia, Tanzania and South Africa), Europe (UK, Ireland, Ukraine, Hungary, Sweden, Russia, Slovakia and Czech Republic), Asia (Israel, Iran, India, China, Sri Lanka, Bangladesh, South Korea, Indonesia, Malaysia and Philippines), Oceania (Australia and New Zealand), North America (USA and Canada) and South America (Brazil, Colombia and Ecuador) (Mohamed *et al.*, 2022). However, the trials have focused more specifically on secondary prevention (Rao *et al.*, 2022). The only RCT so far well powered enough to evaluate the clinical outcomes of using the Polypill for population-wide primary prevention showed ∼40% reduction in the risk of major cardiovascular events (including heart attacks and strokes) in adults (aged 40–75 years) without a history of cardiovascular disease followed up for 5 years (Roshandel *et al.*, 2019).

As is very often the case with innovations (Hughes, 2011; Castellion and Markham, 2013; Abimbola and Patel *et al.*, 2019), in the 20 years since the idea was birthed, the Polypill is yet to be widely adopted and scaled up. This is despite ongoing efforts by ‘researcher-entrepreneurs’—i.e. by researchers and their associates who have sought to generate evidence on the Polypill’s efficacy and have, over time, worked as policy and practice entrepreneurs to institutionalize the innovation. It is therefore important to understand, from a governance perspective, strategies that have been used, considered and proposed over time by these researcher-entrepreneurs to institutionalize the Polypill in the face of uncertainties. The research question was then as follows: how have efforts over time to optimize the adoption and scale-up of the Polypill worked so far? In answering the research question, our primary aim was to develop and test a framework that would be useful for actors involved in introducing health system innovations and promoting their adoption and scale-up. In addition to the Polypill, the insights so generated can, in turn, help policy and practice entrepreneurs anticipate and surmount obstacles for other innovations.

## Methods

We applied a realist-informed approach to our analysis. We first theorized ‘context’ and ‘mechanism’ from the literature on entrepreneurial action in the face of institutional uncertainty; and we then used empirical data to operationalize the theorization (Abimbola *et al.*, 2016; 2019; 2022; Braithwaite *et al.*, 2018; Shaw *et al.*, 2018) The premise of realist analyses is that ‘outcomes’ (in this study, adoption and scale-up of an innovation) of social action are generated through human agency (in this study, entrepreneurial effort) within certain structures (in this study, existing institutions). These structures are referred to as ‘context’, while the manifestation of human agency is referred to as ‘mechanism’. Interactions between context and mechanism generate outcomes—i.e. adoption and scale-up of the cardiovascular Polypill. What we sought to do was understand, through qualitative process tracing (Skarbek, 2020), the ways in which existing institutions (context) influence how (mechanisms) policy and practice entrepreneurs went about their efforts to promote the adoption and scale-up of the cardiovascular Polypill.

### Theoretical framing

**Figure 1.**
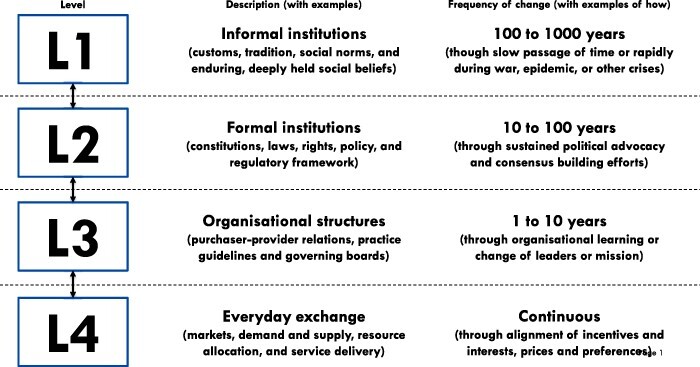
The four levels of institutions, their examples, frequency of change and how change tends to happen (adapted from Williamson, 2000)

This study was informed by two frameworks, although one framework was an elaboration of the other. We combined both frameworks to form what we refer to as the ‘four-by-four’ framework. The first framework, developed by Williamson (2000), recognizes four types/levels of interacting rules (or institutions) that characterize governance within or across settings. It also theorizes how frequently institutions at each level change or how readily they respond to efforts to change them (Williamson, 2000)—see Figure 1. Level 1 (L1) contains the norms and culture of society (informal rules such as customs, traditions and codes of conduct) theorized to change very infrequently—between 100 and 1000 years, and with much inertia, except during or in the aftermath of crises (such as wars, epidemics and social upheaval); Level 2 (L2) is made up of government regulations and policies (formal rules such as constitutions, regulations and policies) theorized to change more readily, but still quite infrequently—between 10 and 100 years; Level 3 (L3) consists of organizational governance (structures such as the nature of governing boards, contractual relations and social mission) theorized to change more frequently—between 1 and 10 years and Level 4 (L4) consists of everyday demand and supply feedback relations (such as those involved in service delivery relations) theorized to change continuously (Williamson, 2000).

The second framework, proposed by Bylund and McCaffrey (2017), elaborates on two main implications of Williamson’s four levels from the perspective of entrepreneurial action. The first implication is that there is greater ease of adoption and scale-up when rules at all four levels are aligned. When prevailing customs and norms in a society (L1) facilitate the development, implementation and enforcement of formal rules (L2) that are conducive to the adoption and scale-up of an innovation, these formal rules facilitate organizational disposition (L3) to develop and deliver the innovation, which in turn ensure that its delivery is well resourced and responsive, supply is available and the price is right to optimize adoption and scale-up (L4) (Bylund and McCaffrey, 2017). In this formulation, higher-level institutions enable or constrain what is possible at lower levels. The rules at immediately higher levels (e.g. L2) determine what is possible at the next lower level (e.g. L3) by determining how lower-level institutions are ordered. The state of affairs at a lower level may also, through feedback, influence institutions at the immediately higher level (Bylund and McCaffrey, 2017).

The second implication drawn by Bylund and McCaffrey (2017) follows from the first. When institutions are well aligned at all the four levels, entrepreneurial efforts ‘abide’ with existing institutions. However, when institutions are not well aligned, and there is institutional uncertainty, entrepreneurial efforts may seek to ‘evade’ or circumvent the influence of the misaligned institution—e.g. by disguising or camouflaging the innovation so that it appears more amenable to existing institutions or by moving to a different location where existing intuitions are better aligned, less uncertain or more conducive. In the context of institutional uncertainty, entrepreneurial action may also consist of efforts to ‘alter’ the misaligned institution—e.g. by advocating for policy change at L2 or restructuring or forming a new organization at L3—while recognizing that successfully altering an L1 institution is highly improbable. Another option for entrepreneurs is to exit when institutional uncertainty constitutes an obstacle to their efforts to promote the adoption and scale-up of an innovation—e.g. by abandoning the innovation or developing a new one. Hence, there are four options available to entrepreneurs—abide, evade, alter and exit (Bylund and McCaffrey, 2017).

### Data collection

S.A.—who had not been involved in any Polypill work but has followed its evolution since the late 2000s—drafted an initial set of interview question prompts adapted from a previous study (Abimbola and Patel *et al.*, 2019) based on domains of the NASSS framework. H.L.—who had conducted process evaluations of Polypill trials since the early 2010s—reviewed these prompts in discussion with S.A. Following these discussions, S.A. and H.L. refined the draft questions to construct the ones below, as interview prompts for qualitative data collection:

- What do you understand about the condition for which the Polypill was developed? (Example of further prompt: How did social, cultural or economic factors influence decisions about primary and/or secondary prevention or cardiovascular disease?)
- What did/do you think about the Polypill strategy? (Example of further prompt: How well was it—or could it be—integrated within the health-care system, and were there any concerns about the use of the Polypill—if there were, what were they?)
- What did clinicians and patients think of the Polypill strategy? (Example of further prompt: To what extent did clinicians think it would help them do their job better, and to what extent did patients think it would help them manage their condition better?)
- What do you think about the economic aspects of the Polypill strategy? (Example of further prompt: What was the business case for developing the strategy, and was there, at the outset, an idea for how the uptake and rollout would be funded?)
- What explained differences in effectiveness, adoption and scale-up between sites/countries? (Example of further prompt: What aspects of context in a country do you think influenced the extent to which they adopted or scaled up the Polypill compared to another country?)
- What factors in the wider environment do you think affected the uptake of the Polypill strategy? (Example of further prompt: Do you think there was a policy push or policy resistance to this strategy—whether at global, national or subnational levels?)
- To what extent do you think the Polypill strategy will be sustained over time? (Example of further prompt: What do you think are the pressures on the horizon—and do you think any of these will affect the use or promotion of the Polypill strategy?)

We conducted interviews—all remotely—between November 2020 and April 2021 with 13 researcher-entrepreneurs who have had various roles in the course of the evolution of the cardiovascular Polypill. Initial interviewees (eight) were identified based on their record of published research and advocacy on the Polypill. Additional interviewees (five) were identified through snowball sampling, with referral from initial interviewees, based on past and current involvement of the individuals being refereed (as researchers and/or entrepreneurs) in efforts to promote the adoption and scale-up of the cardiovascular Polypill.

Of the 13 interviewees, 10 were researchers who have conducted Polypill trials, sought to promote its adoption and scale-up by setting up companies, advocating to global organizations, to national and subnational governments and to health-care organizations and professionals—and/or supported others to do some or all of the preceding. The other three interviewees were non-researchers who have worked in (for-profit and non-profit) organizations supporting researchers’ efforts to promote the Polypill. The efforts of all 13 interviewees spanned LMICs and HICs: five were female and eight were male, with seven currently involved and six previously involved in the Polypill’s adoption and scale-up. For ethics reasons, their specific identities and locations are anonymous—see Table 1 for information on each interviewee’s involvement and duration of involvement in Polypill efforts.

**Table 1.** Interviewee characteristics

ID	Involvement in efforts to promote the adoption and scale-up of the cardiovascular Polypill	Duration of involvement (years)
1	Academic researcher involved in designing and conducting clinical trials on the cardiovascular Polypill and FDCs in Asia, Oceania and Africa and in national and global advocacy to promote their adoption and scale-up	10–15
2	Academic researcher involved in analysing the results of clinical trials on the cardiovascular Polypill and FDCs in Oceania—with a focus on the social and economic aspects of their adoption and scale-up	5–10
3	Academic researcher involved in designing and conducting clinical trials on the cardiovascular Polypill and FDCs in Asia—with a focus on studying the social and economic aspects of their adoption and scale-up	5–10
4	Academic researcher and clinician involved in designing and conducting clinical trials on the cardiovascular Polypill and FDCs in Asia, Oceania and Europe and in national and global advocacy to promote their adoption and scale-up	15–20
5	Academic researcher and clinician involved in designing and conducting clinical trials on the cardiovascular Polypill and FDCs in Africa and North America and in national and global advocacy to promote their adoption and scale-up	10–15
6	Academic researcher involved in designing and conducting clinical trials on the cardiovascular Polypill and FDCs in Asia, Oceania and Europe and in national and global advocacy to promote their adoption and scale-up	15–20
7	Academic researcher and public health practitioner involved in the regulatory governance of new drugs in Oceania and in the governance of organizations involved in national and global advocacy to promote the adoption and scale-up of the cardiovascular Polypill and FDCs	5–10
8	Business executive of organization involved in financing clinical trials on the cardiovascular Polypill and FDCs and in national and global efforts to promote their adoption and scale-up	5–10
9	Academic researcher and clinician involved in designing and conducting clinical trials on the cardiovascular Polypill and FDCs in Asia, South America and Europe and in national and global advocacy to promote their adoption and scale-up	10–15
10	Academic researcher involved in designing and conducting clinical trials on the cardiovascular Polypill and FDCs in Africa and in national and global advocacy to promote their adoption and scale-up	5–10
11	Business executive of organization involved in financing clinical trials on the cardiovascular Polypill and FDCs and in national and global efforts to promote their adoption and scale-up	5–10
12	Business executive of organization involved in financing clinical trials on the cardiovascular Polypill and FDCs and in national and global efforts to promote their adoption and scale-up	5–10
13	Academic researcher and clinician involved in designing and conducting clinical trials on the cardiovascular Polypill and FDCs in Europe	5–10

The questions listed earlier were used as prompts in a conversational interview. All the interviews were conducted in English. Each participant was invited to tell the story of their involvement in the Polypill adoption and scale-up in their own words and reflect on the multiple interacting influences on it. Each interview lasted about 1 h, was audiotaped with consent and professionally transcribed. Both authors jointly conducted all the interviews except one.

### Data analysis

Each interview was followed by a 15- to 30-min debrief session in which both authors discussed the conversation, reflected on which strategies the interviewee had implicitly used, to what end, at what level of institutions and how these changed over time in their effort to promote the adoption and scale-up of the Polypill. Each author took notes or made mental notes of how subsequent interviewees aligned or diverged in their perspectives, efforts and goals—and what belonged under each category of strategy and of levels of institutions. This was refined and adjusted with each interview. In this process, both authors agreed to have reached saturation after the 13th interview; unsurprising, given that our aim was to sample from a broadly homogenous and internationally networked group of researcher-entrepreneurs. In the realist analysis, we sought to explain passages of transcripts in which interviewees assessed the state of adoption and scale-up (i.e. ‘outcomes’) due to their or others’ entrepreneurial effort (i.e. ‘mechanism’, categorized as abide, evade, alter and/or exit) in terms of the institutions (i.e. ‘context’, categorized as L1–L4) that enabled or constrained entrepreneurial action.

As the analysis proceeded, we made repeated references to previous and subsequent passages within each transcript and to parts of other interview transcripts. In doing so, we explored and sought to reconcile divergent narratives and the assumptions underpinning adoption and scale-up efforts. Factual errors (e.g. on the findings of specific studies or on timelines) were corrected by referring to the literature on the Polypill, which had been authored by either the interviewees or their collaborators. A provisional summary of findings was produced by S.A. and then discussed with H.L. Identified categories were debated, refined and adjusted by S.A. and H.L. until there was a coherent scheme that broadly accounted for the range of entrepreneurial action and institutions. This stage of the analysis included a search for disconfirming data from the interviews that might challenge our emerging interpretation—to ensure that we were not cherry-picking quotes and explanations that supported our interpretation. Phrases or quotes that most aptly expressed or illustrated the findings were then identified. In the Findings section, the number (from 1 to 13) in brackets after each quote corresponds to the particiant ID listed in Table 1.

### Ethics

Ethics approval for data collection was obtained from the University of New South Wales Human Research Ethics Committee (approval #: HC200174). Participation in the interviews was voluntary, based on the participant signing an informed consent form. In line with the terms of consent, interview data are not publicly available and have been de-identified in this report, by removing potentially identifying information such as name, gender, location, employer and specific nature of work. The participants are described broadly as ‘researcher-entrepreneurs’. In the analysis, findings were abstracted to the level of entrepreneurial strategies, and locations were abstracted to the level of HICs and LMICs without naming countries. The de-identified data are available on reasonable request.

## Findings

**Figure 2.**
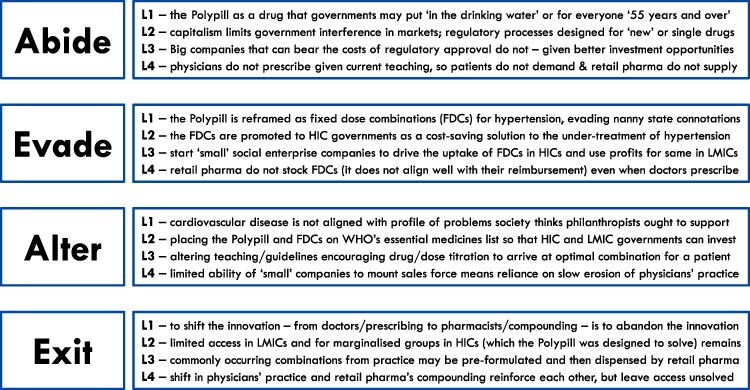
Illustrating the institutions at each level and the strategies used or considered by researcher-entrepreneurs in promoting the adoption and scale-up of the cardiovascular Polypill

**Figure 3.**
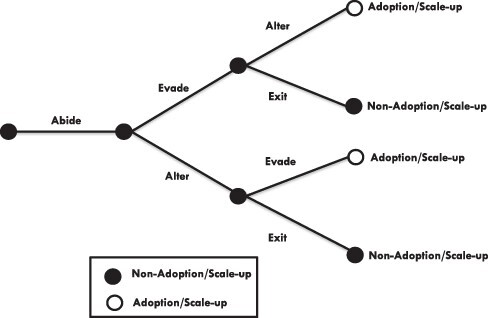
The unfolding of four strategies used or considered concurrently by researcher-entrepreneurs in promoting the adoption and scale-up of the cardiovascular Polypill

The analysis revealed shifting and overlapping strategies that began with efforts to abide or work within existing institutions (see Figure 2). When they failed, efforts to abide were followed by efforts to evade and/or alter misaligned institutions—not so much sequentially, as was concurrently (see Figure 3). The results of (anticipated) failure of efforts to abide, evade or alter were then followed by suggestions and efforts to exit entrepreneurial effort for the Polypill altogether. The shifts in participants’ thinking from one strategy to another, and their overall reconsideration of strategies, were propelled by ‘learning’.

The initial plan was to promote the Polypill in both HICs and LMICs, with three broad sets of theories of change. First, there was a sense that in HICs, the Polypill will help address an intractable problem of physician reluctance to prescribe multiple drugs at the same time, especially early in the course of treatment—given that the current practice is for physicians to titrate over time to achieve the optimal combination of drugs and doses. Second, by improving medication adherence (given the greater ease of use), the Polypill will benefit underserved and marginalized groups in HICs for whom there was a perception that by reducing pill burden, the Polypill will enhance medication adherence—because of their complex life circumstances that may otherwise limit adherence. The third theory of change was that the Polypill will generally benefit people in LMICs, with the expectation that the significantly reduced cost of medication (if it consists of generic medicines) means that the Polypill will not only improve physicians prescribing behaviour and reduce pill burden but also increase access. Notably, among the researcher-entrepreneurs interviewed, the main aim for promoting the Polypill was for secondary prevention—and not primary prevention as was initially advocated by Wald and Law (2003).

### Abide

Efforts began by abiding with existing institutional arrangements governing the adoption and scale-up of medicines. The researcher-entrepreneurs began by showing (through their RCTs) the Polypill’s efficacy in improving prescription practices, medication adherence and risk factor control. There was less focus on first studying the workings of the health systems into which the Polypill would be introduced in order to understand the institutions to abide with and the Polypill’s potential for scale-up to reach people who needed it—or to examine whether they needed it, or what they might need instead: ‘if you set the scene more thoughtfully at the start, then you’re potentially going to have a better uptake’ (2) ‘… rather than the process that was taken … where we did the study [i.e. RCT] and then we did the process and economic evaluation towards the end’ (2). Efforts to promote the Polypill’s uptake revealed perceptions (independent of the evidence) that constrained its adoption and scale-up. At L1, there was a legacy issue linked to its initial framing as a drug for everyone aged ≥55 years, that governments may put ‘in the drinking water’ (13). Although the Polypill being promoted was not for universal use (but rather for use among people with or at high risk of cardiovascular disease), many (including physicians and patients) considered it potentially so. Another L1 constraint was the entrenched societal perception of cardiovascular disease as a lifestyle condition and so does not deserve social investment (whether by governments or philanthropists) in an innovation or radical change in medical practice.

Early on at L2, it became apparent that the institutions of free-market capitalism (i.e. government non-interference in markets) were a constraint—i.e. even if the Polypill was effective, governments may be constrained in their ability to compel pharmaceutical companies to make it available or affordable. Another L2 constraint was that regulation of pharmaceutical products is optimized for concerns around safety (to avoid toxic products getting to the market) and evergreening (to avoid repurposing existing drugs to extend their patent periods). Although the Polypill was a combination of existing drugs that patients took together, it had to undergo an approval process like a new drug, as it was being proposed for a new indication—for untreated people who may not be eligible for all the drugs in the combination. The rules were described by respondents as ‘10, 15, 20 years old’ (6), change ‘takes many years’ (6), ‘not a lot of flexibility’ (7), ‘didn’t have any latitude’ (6) and ‘if you’ve got something a little different, you’ve got to shoehorn your development into their pathway’ (6). While seeking regulatory approval, researcher-entrepreneurs ran into a problem—aspirin in the Polypill was not bioequivalent to when taken alone.

Given L2 constraints, the response of L3 actors to the Polypill was suboptimal—‘it’s quite expensive for a company to bring forward [a regulatory] application; they’ve got to have a pretty good sense of what the market’s going to be to want to do it’ (7). The HIC market was seen as limited to the poor—and big pharmaceutical companies were not interested in a drug for disadvantaged populations. Although the LMIC market is large, it is deemed commercially better to invest in a product with large marginal profits per patient even if the patient population is small—than investing in a product (such as the Polypill) with potential for only small marginal profits per patient even if the patient population is large. Efforts to persuade big pharmaceutical companies failed repeatedly. Small and generics pharmaceutical companies lacked the financial capacity or determination to surmount L2 regulatory hurdles—many tried and later gave up, one after the CEO who launched the effort left, ‘which happens every five or 10 years, regularly’ (6). The interest from pharmaceutical companies waned because there was a lack of assured purchase by HIC governments or international agencies on behalf of LMIC populations (as may occur for diseases that threaten global health security, for which governments may override L2 institutions of free-market capitalism to demand that companies make drugs available, and at a price that is affordable). Cardiovascular disease was not deemed worthy of such exceptions. As a set of L3 institutions, established teaching, practice and guidelines that prescribe titrating drugs and doses over time persisted as the dominant paradigm. As a result, physicians—at L4—who would otherwise prescribe the Polypill were reluctant to do so.

In addition to L3, the perception of the Polypill at L1 and the constraints at L2 affected its adoption at L4—i.e. the interface among physicians, patients and retail pharmacies. If physicians do not prescribe it due to their perception of its safety or the persistence of established practice or because it contains off-label generic ‘drugs that are considered outdated’ (3), retail pharmacies will not stock it. If patients do not demand it due to its costs and affordability, or the perceived low quality of its generics content or its intrusive connotations, then physicians will not prescribe it. If pharmacies do not stock it due to weak supply chain because of uncertainties about demand, poor patent perceptions or lack of government subsidy, then physicians will not prescribe it. The Polypill was caught in this L4 vicious cycle.

### Evade

The failure of the ‘abide’ strategy led to a strategy to ‘evade’ institutional obstacles—‘if we’ve learned anything, it was that the Polypill was a paradigm change too far, and to iterate more slowly’ (6). Evade entailed horizontal repositioning by researcher-entrepreneurs. They shifted from promoting the Polypill to promoting fixed-dose combinations (FDCs) containing two or three anti-hypertensive drugs—anti-hypertension drug combinations marketed by big pharmaceutical companies were already in use. They shifted from promoting the Polypill across both HICs and LMICs to promoting it in selected HICs—where public and private entities could pay. The evade strategy took the form of a social enterprise. The profits from successful adoption and scale-up of FDCs or the Polypill in HICs would then be used to underwrite the Polypill in LMICs. Now an FDC, the purpose of the innovation became primarily to address the inertia of physicians to prescribe multiple drugs early in during treatment. This strategy evades an L1 obstacle—the innovation could now no longer be mistaken for the population-wide version of the Polypill.

Hypertension FDCs evade L2 constraints by avoiding the Polypill’s manufacturing challenges. Even then, researcher-entrepreneurs had to ‘follow the rulebook of regulatory guidelines’ (6). Evading L2 obstacles also involved reframing problems that FDCs could solve as problems that HIC governments may consider high priority—as under-treatment of millions of their citizens with hypertension, a problem to be solved with urgent government investment in hypertension FDCs to reduce the costs of strokes, heart attacks and early deaths from under-treated hypertension, especially among structurally disadvantaged groups (people of low socio-economic status or in rural and remote communities and ethnic minorities). But the link between reduction in pill burden and improved adherence on population health outcomes is difficult to show empirically. For chronic diseases, there can be a ‘forgiveness period’ (7) in which skipping doses or delaying the start of treatment may be inconsequential. The impact of pill burden or frequency may also depend on the size of reduction—two/twice to one/once less than say three/thrice to one/once. More than changing the regimen, improving population health outcomes may also require getting more people diagnosed and on treatment, a problem that hypertension FDCs (or the Polypill) cannot address, unless if administered at a certain age regardless of diagnosis, as was the original idea of the Polypill.

Evading L2 constraints involved creating or seeking new L3 institutional forms, companies that function at a scale between a big pharmaceutical company (with capacity to see a new drug through regulatory or marketing processes) and a generics company (with limited capacity). Operating as social enterprises, these companies ‘will put the pills through regulatory trials, get approvals, and probably partner with [big] pharmaceutical companies to get it out into the market’ (1) or ‘get eaten up [i.e. acquired] by a big company that’s got a huge distribution network’ (5). But pricing remained an issue. In many HICs, the price of blood pressure medication is already low, so FDCs may be perceived as expensive by payers, given the price increase associated with the cost of combining already affordable drugs. Another relevant L3 institution was the model of purchaser–provider relations. If an insurer doubles as service provider, it is easier to make the case to invest in prevention through the FDCs (or the Polypill) because the insurer picks up the costs of complications. Another L3 institution was how retail pharmacies are reimbursed. If reimbursed per number of drugs dispensed, retail pharmacies are less disposed to stocking FDCs. Hence, researcher-entrepreneurs sought settings with favourable purchaser–provider and reimbursement arrangements. But similar to the ‘abide’ scenario, an L3 institutional hurdle (current training, practice and guidelines) meant physicians were still likely to be reticent to prescribe FDCs.

The combined effects of L1, L2 and L3 constraints on L4 meant the evade strategy was only marginally effective. The vicious cycle remained. Physicians remained committed to finetuning dosage and choice of medicines for patients. Retail pharmacies did not stock supplies reliably, in part because FDCs were not well aligned with their reimbursement structure. Purchaser–provider relations were not optimized for prioritizing preventive interventions. Having a research programme on how the constraints interact in various settings was seen as essential, especially as institutional obstacles persisted in the face of the FDC research programme on effectiveness trials across different settings. The growing focus of efforts on the evade strategy also raised concerns among researcher-entrepreneurs that ‘we are almost slightly losing the original grand idea’ (12) of improving access to medicines in LMICs through the Polypill.

### Alter

The evade strategy provided a lesson—that it might be necessary to alter existing institutions from L1 to L4 in HICs and LMICs—for both the Polypill and hypertension FDCs. Altering L1 institutions or their effects involved persuading philanthropists to fund the innovation. However, societal norms around the type of problem that philanthropists (should) solve or the solutions they (should) support proved immutable—‘heart disease didn’t really tick the box for philanthropy’ (6) or ‘well, these are chronic things, we got to fix some other stuff first’ (5), especially those other health problems that are ‘emotionally engaging’ (6) or tug at ‘heartstrings’ (6), with ‘get-rid-of-the-problem’ (6) solutions like human immunodeficiency virus/acquired immune deficiency syndrome or child mortality. Cardiovascular disease did not fit the profile. Unlike what the Polypill and hypertension FDCs could offer, philanthropists were ‘very attracted by definitive solutions, cures … moon-shot innovations’ (6). These distinctions became clearer for researcher-entrepreneurs who sought to persuade philanthropists.

Altering L2 constraints involved tackling government unwillingness or inability to pay for the Polypill and hypertension FDCs by lobbying the World Health Organization (WHO) to place them on the essential medicines list of countries around the world. The researcher-entrepreneurs hoped that this would make national governments and international agencies more willing to pay for them—and inspire confidence among pharmaceutical companies (to invest in), physicians (to prescribe) and patients (to demand) the drugs. It took almost 10 years of sustained advocacy to secure WHO’s approval. Even so, there was a sense among researcher-entrepreneurs that regardless of WHO’s approval, LMIC governments and international agencies had not yet chosen to invest in or support the drugs. Inertia remains too among HICs to tweak their regulatory or funding rules, given uncertainty about the potential of drugs to improve population health outcomes. Researcher-entrepreneurs came to accept that the Polypill and hypertension FDCs may not be enough to improve medication access and affordability or reduce the burden of untreated and poorly controlled high blood pressure in both HICs and LMICs.

Altering L3 constraints involved educating and socializing physicians to alter current teaching, practice and guidelines about titration of cardiovascular medications—‘That really is a paradigm shift and that has to start in medical schools, and it may take a generation’ (4); ‘We have to go against a century of medical education’ (13). There is an ongoing, but slow attrition of this constraint, as researcher-entrepreneurs repeatedly present evidence (in academic journals or conferences) from RCTs on the benefit and safety of the Polypill or hypertension FDCs, and as big pharmaceutical companies market their own two-drug hypertension FDCs at academic conferences and through their sales force. While big pharmaceutical companies did not market their FDCs to reach underserved populations, their marketing helped researcher-entrepreneurs’ efforts in socializing the Polypill concept. Physicians were more receptive to two-drug FDCs than the full Polypill, and so this was a fitting starting point to alter entrenched practices—a lesson carried over from the evade strategy. But there remains a snag in that physician opinion leaders in HICs—i.e. influential academic cardiologists at elite HIC centres—have ‘an impenetrable belief that Polypills are a second-class solution for LMICs’ (4). In spite of the evidence, many believe that HIC health systems are so well financed and they do not need a ‘blunt instrument’ (4) like the Polypill. Another lesson learnt from the evade strategy was that altering L3 constraints from purchaser–provider split required demonstrating the adoption experience of purchaser–providers (e.g. government agencies and insurers) rewarded for controlling patients’ blood pressure.

Altering L4 constraints involved changing the perceptions of patients who equate generic to low quality by rebranding the Polypill ‘using the word affordable rather than cheap’ (11) or ‘creating a new name for it as a new whiz-bang drug’ (2). Altering L4 constraints also involved changing physician behaviour at the point of prescription which then influences patient preference. But generics companies do not have the resources of big companies: ‘Generics don’t have big sales forces … [but] you need someone whose job it is to remind you of that evidence all the time and put it in front of your face’ (4); ‘Well, who’s going to pay for that? If your drug has got next to no margin, who’s going to support that?’ (6). While the slow erosion of entrenched physician practice continues to shift the prescribing landscape in HICs, this shift does not solve the more LMIC problem—i.e. getting governments and businesses to make the Polypill and hypertension FDCs available in retail pharmacies. Altering L4 constraints also require changing the reimbursement structure of retail pharmacies (L3), so they are incentivized to stock the medicines.

### Exit

Another option revealed in the reasoning of some researcher-entrepreneurs was to exit. For them, to exit was to recognize that perhaps the Polypill concept was too ambitious or that it was time to try a new premise, e.g. by having the physician prescribe the cardiovascular medications as normally done, and if requested by the patient, a pharmacist can then dispense the medications as a polypill:

*I would change the term ‘Polypill’ because it has a link with the original concept, which I think is far-fetched. If you talk about the Polypill, people say: ‘We’re not going to do that’, because they think that it’s in the drinking water and that’s not the idea. But … my take on it is that … the physician puts people on cardiovascular prevention [medications] and then settles that to a certain level in which patient and physician are happy. Then they go to the pharmacy, and they say: ‘Hi you guys, put it in one tablet.’* (13)

The dispensing approach to the Polypill can address the problem of adherence and preserve physicians’ ability to decide what goes into each combination, while making institutional constraints at L1 and L2 irrelevant. At L2, exit removes the need to evade or alter constraints imposed by free-market capitalism—i.e. national governments and international agencies do not need to take any special action. While L1 constraints on acceptability of ‘the Polypill’ can be removed by ‘exit’, L2 constraints on access remain. But the Polypill may not be best placed to address access—‘Polypill doesn’t answer the bigger problem around getting medications to the patients … that’s the thing to solve. If you can solve that, you’ve got low-cost medications… The biggest challenge is access’ (11). Framing the Polypill as a means to improve access may have distracted from or crowded out more appropriate solutions. Researcher-entrepreneurs widely acknowledged that they had focussed more on the solution (the Polypill) than on the problems it could (not) solve, or on those it might exacerbate, or may be better solved without it—e.g. convincing physicians to prescribe a combination of drugs early in the treatment process in a way that preserves a physician’s ability to decide alongside their patient which drugs go into each combination.

The exit strategy builds on gains made in the ‘alter’ strategy at L3 to change current teaching, practice and guidelines. Endorsed by the WHO, the Polypill and hypertension FDCs are increasingly recommended in clinical guidelines. The practice of titrating drugs and doses is changing, but more among cardiologists who have been the primary target of training, advocacy and marketing—by researcher-entrepreneurs and big pharmaceutical companies. With training offered to medical students, general practitioners and other specialists, this shift may be quicker, without the concerns of inflexible prescription associated with the ‘fixed’ Polypill or hypertension FDCs. Exit preserves the possibility of ‘Polypills’—note, plural—i.e. commonly occurring combinations that emerge from practice may then be pre-formulated. Such ‘individualised Polypills’ (13) remain flexible to the emergence of new, more effective drugs; ‘if people develop side effects, you can change the drug and then see what happens’ (13) and ‘there is not one single combination pill … we get a variety of … combinations, unless you make a certain number of established buyable single combinations … that are comprising 40 percent of the market’ (13).

The exit strategy can break the L4 vicious cycle, by making the Polypill more acceptable ‘as a concept’ to prescribing physicians—and to dispensing retail pharmacies who may no longer lose income if they no longer have to dispense the Polypill as a prescribed single medication. Patients may not need to request the Polypill from physicians, but instead ask of their physician or retail pharmacist that their drugs be dispensed within single capsules. As advocacy and the results of RCTs continue to chip away at physician objections to prescribing combinations of drugs early in the course of treatment, exit might be well suited to wide adoption and scale-up of this new practice. Some retail pharmacists may need additional training or equipment for this mode of dispensing which ‘for lower income countries it may be more difficult, but for [HICs], there should be little or less problems’ (13). Even among those who did consider this exit option, there was a sense that having other adherence-improving innovations—such as ‘long-acting injections’ (5)—may compel another exit or serve as a more compelling rationale for exit.

## Discussion

In line with existing theorization, at different stages, researcher-entrepreneurs used or considered four modes of functioning in relation to existing institutions: abide by them, seek to evade or alter them or exit from entrepreneurial action (Hirschman, 1970; Oliver, 1991; Bylund and McCaffrey, 2017; Elert and Henrekson, 2017). What had not been as clearly theorized is how one response leads to another; when it does, what mechanisms enable such shifts in strategy. Our findings suggest that one such mechanism is learning (Weick, 1984; Fiol and Lyles, 1985; Schon, 1994) (i.e. making the link ‘between past actions, the effectiveness of those actions, and future actions’; Fiol and Lyles, 1985) or the progressive ‘turning of hindsight into foresight’ by researcher-entrepreneurs. In our study, learning occurred primarily through ‘action’, one of the three ways through which learning occurs in health systems as described by Sheikh and Abimbola (2021). The two other ways are through ‘information’ and ‘deliberation’ (Sheikh and Abimbola, 2021). Learning through action involved the use of information and deliberation (e.g. information from RCTs and feedback from stakeholders) but less use of deliberation (with patients, physicians, pharmaceutical companies, philanthropists, governments and international agencies) to anticipate and prevent inevitable obstacles to adoption and scale-up.

A question worth asking therefore is how much of what was learnt as events unfolded could have been anticipated from the beginning with a different set of priors about what it would take to promote the adoption and scale-up of the Polypill? To answer this question, it is important to consider what has been theorized as three loops of learning (Argyris and Schön, 1996; Tosey *et al.*, 2012; Sheikh and Abimbola, 2021). With single-loop learning, the learner adjusts their action. With double-loop learning, the learner adjusts the rationale for their action. With triple-loop learning, the learner sets up structures that will facilitate single- and double-loop learning in the future (Argyris and Schön, 1996; Tosey *et al.*, 2012; Sheikh and Abimbola, 2021). Much of the learning from entrepreneurial action in this study was single-loop learning. But there was a slow accretion of double-loop learning that made some researcher-entrepreneurs to consider the exit option or to question their rationale—i.e. to question the Polypill or hypertension FDCs as optimal solutions to access, affordability or population prevention of cardiovascular disease. Triple-loop learning means setting up structures or developing frameworks to help put hindsight in the service of foresight. While we did not find evidence of triple-loop learning in the Polypill adoption and scale-up story, we hope that the four-by-four framework we have proposed may facilitate such triple-loop learning in the future.

**Figure 4.**
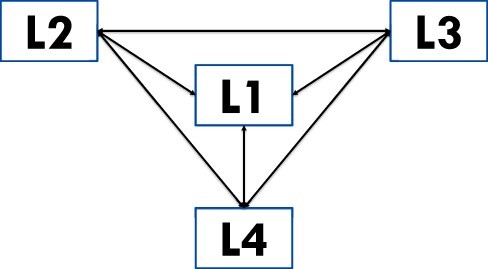
The four levels of institutions rearranged so that all have bi-directional relations with one another

Our findings suggest two modifications to this framework that juxtaposes four levels/types of institutions with four entrepreneurial options available to policy and practice entrepreneurs seeking to promote the adoption and scale-up of innovations in the face of institutional uncertainty (Williamson, 2000; Bylund and McCaffrey, 2017). First is a recognition that the options can be tried out sequentially or even concurrently by researcher-entrepreneurs working on the same or similar innovation (see Figure 3). The three other options came after trying or failing at abide. Policy and practice entrepreneurs may be well served to lay these out as potential options from the start. Second, the four levels/types of institutions were arranged such that only adjoining levels/types of institutions could influence each other directly (Williamson, 2000). However, we found that L1 (e.g. societal perceptions) may influence all the other levels directly and that L2 (e.g. government intervention) may influence L4 directly and vice versa (e.g. via demand and supply relations). It may thus be more fitting to rearrange the levels/types of institutions such that L2, L3 and L4 are interconnected bidirectionally in a ‘triangle of rules’ (Abimbola, 2020), with L1 connected bidirectionally to all three nodes of the triangle (see Figure 4).

Our findings are in line with the literature showing the dynamic nature of innovations—as they take on new forms during their evolution, in response to shifting demands, changing technology or institutional uncertainties (May and Finch, 2009; Greenhalgh *et al.*, 2017a). Our findings are also in line with evidence on the Polypill (Webster *et al.*, 2017; 2020; Khan *et al.*, 2023) and other innovations (Abimbola and Keelan *et al.*, 2019; Abimbola and Patel *et al.*, 2019; Bulthuis *et al.*, 2020) that understanding how to align interests and create the perception at multiple levels that the benefits of an innovation outweigh its costs (politically and economically) are essential for adoption or scale-up. Efforts to promote the adoption and scale-up of the Polypill continue. The evade and alter strategies remain viable—even if success is slow, and the future is uncertain. Even the exit option in our study does not suggest a complete letting go of the innovation. It is just an acknowledgement that if adoption and scale-up of the Polypill was to happen, it may have to go through a different route, not in the same way, to the same extent, or to solve problems as initially anticipated. This exit strategy may even be considered a form of evade. And the evade strategy—i.e. rebranding the Polypill as FDCs—may be described as exit. Just as entrepreneurial efforts to promote using the Polypill for secondary prevention represents an exit from its primary prevention branding—even as studies were being conducted to provide evidence for using the Polypill for primary prevention (Roshandel *et al.*, 2019). As such, these four options are open to how an analyst interprets the nature of the actions involved under each option (Oliver, 1991).

This study is inevitably incomplete. What we have done is to isolate how two important elements in the adoption and scale-up of the Polypill intersect—entrepreneurial strategies and institutional context. In the process, we also identified the crucial role that learning plays at that intersection. But there are other important elements involved—e.g. what types of evidence matter for each category of Polypill stakeholders, or the relations between researcher-entrepreneurs and other Polypill stakeholders, the perspectives of Polypill stakeholders other than researcher-entrepreneurs, the veracity of researcher-entrepreneurs’ working assumptions about the Polypill’s potential, the role that the social and quality connotations of generics drugs to different stakeholders played in the acceptance of the Polypill, the unfolding of the evolution of attitudes towards the Polypill in specific countries and how attitudes might vary within a country—say, between rural and urban locations, between general practitioners and cardiologists or between socio-economic groups. Each of these elements deserves its own separate study—including analyses of how and under what circumstances the Polypill achieves each of the expectations made on its behalf.

What we are dealing with here—i.e. adoption and scale-up of innovations—is complex, with multiple parts in dynamic interaction with one another. What such complexity demands is that we have multiple accounts from different perspectives and positionalities, using different theories and frameworks (Cilliers, 2004; Appiah, 2017; Bhakuni and Abimbola, 2021). We hope our study inspires such pluralism. Future studies may use the framework of four levels/types of institutions and the four entrepreneurial strategies to explore and compare how other health system stakeholders act in the face of institutional uncertainty while trying to introduce or scale up an innovation. The four-by-four framework should be seen as compatible with existing well-used frameworks such as NPT (May and Finch, 2009; Murray *et al.*, 2010) and the NASSS framework (Greenhalgh *et al.*, 2017a; Abimbola and Patel *et al.*, 2019). Multiple theories can be applied under each domain of the NASSS framework (Greenhalgh and Abimbola, 2019). The four-by-four framework can enrich analysis under domains of the NPT (e.g. ‘coherence’ and ‘collective action’) (May and Finch, 2009) or NASSS framework (e.g. value proposition, institutional and societal context and interaction between domains over time) (Greenhalgh *et al.*, 2017a).

We encourage health system practitioners involved in promoting all kinds of innovation to use the four-by-four framework as a sensitizing tool—to work out, prospectively, what institutional constraints to anticipate, which ones to evade by designing and presenting the innovation differently or which ones to plan to change (and the chances of success at changing them), and the likely circumstances under which exit becomes a sensible option. We also recommend that health system practitioners use the four-by-four framework to document and publish their learning in the course of adoption and scale-up efforts—providing a basis for rich syntheses of lessons across efforts. We encourage researchers to use the four-by-four framework as a scaffold to retrospectively generate rich and contextually situated narratives on why some (and not other) innovations were adopted and scaled up—and to do the same to document the efforts of policy and practice entrepreneurs working across settings to navigate the diverse institutional influences on an innovation. We also encourage researchers to use the framework as a matrix to guide and structure evidence syntheses on the adoption and scale-up of innovations.

## Conclusion

To promote the adoption and scale-up of the cardiovascular Polypill, policy and practice entrepreneurs went through four previously theorized entrepreneurial strategies—abide by existing institutions, seek to evade or alter misaligned institutions or exit entrepreneurial action. The thread going through their efforts over time was learning. The challenge, however, is to ensure that what they learnt is taken as the starting point of future efforts to promote the adoption and scale-up of innovations. We recommend using a four-by-four framework (four types/levels of institutions and four entrepreneurial strategies) as a sensitizing tool to map out contingencies in any effort to introduce or institutionalize an innovation—especially as alignment of institutions at all four levels cannot be taken for granted for most innovations or in most settings. The ‘four-by-four’ framework can be used as a scaffold to generate narratives of previous and ongoing adoption and scale-up efforts or as a matrix to synthesize such narratives and experiences across different innovations and settings. Using the four-by-four framework can optimize transferability of insights generated across efforts and settings to promote the adoption and scale-up of innovations.

## Data Availability

The datasets used and/or analysed during the current study are available from the corresponding author on reasonable request.

## Reflexivity statement

This work was done by a team of two researchers: one male and one female, both early/middle career researchers, both currently based in Australia, but originating elsewhere (one from Africa and one from Asia). This work addresses an issue—the adoption and scale-up of health system innovations, especially under conditions of institutional uncertainty—which poses a challenge globally, but which affects governance settings to be found more in LMICs.
